# In Memoriam Terje Sagvolden

**DOI:** 10.1186/1744-9081-7-5

**Published:** 2011-03-17

**Authors:** Joseph Sergeant, Heidi Aase, Stephen V Faraone, Espen Johansen, Raj Kalaria, Anneke Meyer, Vivienne Russell, Adolfo Sadile, Edmund Sonuga-Barke, Rosemary Tannock

**Affiliations:** 1 Department of Clinical Neuropsychology, Vrije Universiteit, Amsterdam, The Netherlands; 2 The Norwegian Institute of Public Health, Division of Mental Health, Oslo, Norway; 3 Department of Child and Adolescent Psychiatry, State University of New York, Upstate Medical University, Syracuse, New York, USA; 4 Akershus University College, Kjeller, Norway; 5 Institute for Ageing and Health, Newcastle University, Newcastle upon Tyne, UK; 6 School of Health Sciences, University of Limpopo, Sovenga, South Africa; 7 Department of Human Biology, University of Cape Town, Cape Town, South Africa; 8 Department of Experimental Medicine, Second University of Naples, Naples, Italy; 9 School of Psychology, University of Southampton, UK; 10 The Ontario Institute for Studies in Education, University of Toronto, Ontario, Canada

## 

It is with great sadness that we note the sudden passing of our colleague and friend Professor Terje Sagvolden, a highly accomplished neuroscientist, well known across the world for his contribution to our understanding of the neurobiology of attention deficit hyperactivity disorder (ADHD). Here we pay tribute to this magnificent man and scientist in an intercontinental recognition of his contribution to science. Terje was a wonderful caring person, a kind considerate friend and a brilliant researcher. Terje was inspiring and creative, as well as a visionary. He pioneered collaborative research and forged links between basic and clinical researchers in different disciplines, across different countries.

With Terje's help, the European Network for Hyperkinetic Disorders (Eunethydis), of which he was a founding member, obtained a major EU grant in 1993 that enabled the then participating centres to work on the same ADHD project cross-nationally. The effect of this was to harmonize research efforts in several European groups. This forged the basis for the later projects on genetics and intervention evaluation. In the discussions held at Eunethydis meetings, Terje took up the challenge with his characteristic enthusiasm to demonstrate that the animal model he was using had considerable clinical relevance to ADHD.

**Figure 1:**
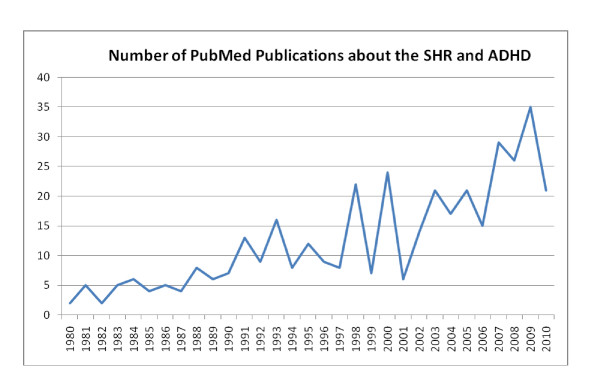
**Number of PubMed Publications about the SHR and ADHD**.

Terje's seminal work was to demonstrate the relevance of the spontaneously hypertensive rat (SHR) for human ADHD. As Figure 1 shows, a PubMed search indicates that from 1980 to 2010, there was an exponential growth of research using the SHR as an animal model of ADHD or hyperactivity.

In 1998 there is the first major spike. This was primarily due to a special issue of Behavioural Brain Research that Terje edited with Joseph Sergeant and contained the particularly important contribution from the Naples group, led by Adolfo Sadile, who Terje met in 1980 in Erice, Sicily. They forged a partnership in the European Brain and Behaviour Society (EBBS) and Society for Neuroscience meetings. Terje became the secretary of EBBS and gave an enormous impulse to the growth of that society. At Eunethydis meetings, the Oslo and Naples groups developed fruitful novel questions, experimental approaches, and produced innovative results, which have been applied to human ADHD.

Animal models in ADHD were something new in the 1980s, hence the challenge on their clinical relevance. By comparing ADHD traits between the SHR and the Wistar Kyoto (WKY) reference strain, Terje was able to establish unequivocally a genetic basis for ADHD traits in the SHR [1], mRNA expression profiles in the brain [2], the genetics of intra-individual variability [3,4] and the effects of methylphenidate on dopamine transporter density [3,4]. Terje made important contributions toward understanding the genetics of ADHD. Because the brains of ADHD patients cannot be studied, some work would not have been possible without a valid animal model of the disorder. Terje validated the SHR as a suitable model of ADHD, although several such models had been proposed, the SHR stands alone it its ability to mimic the fundamental behavioural characteristics of ADHD, conform to a theoretical rationale for ADHD, and predict aspects of ADHD behaviour, genetics, and neurobiology [5-7].

In the premature last phase of his research, Terje made another key discovery that will make lasting contributions to the genetics of ADHD. Although WKY rats had routinely been used as a reference strain without regard to source, Terje showed substantial behavioural and genetic differences in strains from different laboratories [8,9]. This led to the further discovery that WKY rats from Charles River, Germany (WKY/NCrl) showed inattention, but no overactivity or impulsiveness and thus were a suitable model of inattentive ADHD. In contrast, he showed that WKY rats from Harlan, UK were a suitable reference strain for both the SHR and the WKY/NCrl [8,9]. By providing researchers with genetic rat models of both combined-type (SHR) and inattentive (WKY/NCrl) ADHD, Terje will continue to influence the course of ADHD genetics research for decades to come.

Through his work within Eunethydis, he profoundly influenced the work of a younger generation of ADHD researchers, especially through his enthusiastic promotion of reinforcement and learning as an important focus for human ADHD research. This insight has played a seminal role in the fundamental shift in focus from cognitive to motivational models of ADHD that has occurred in the field. In that process of change, Terje took students and new colleagues under his wings and gave them the opportunity to take part in his own research and networks to form their own careers. Terje's ability to be always true to himself was a hallmark of his character.

Africa would like to offer a special tribute to Terje. He was a great friend of the late Professor James Kamani and one of the founders of neuroscience in Africa. He played a significant role in the establishment of the Society of Neuroscientists of Africa (SONA) in 1993. Terje continued to support the promotion of neuroscience in Africa. He served on the International Advisory Committee of SONA and, together with Raj Kalaria, Michael Zigmond, Beth Fischer, Marina Bentivoglio, Anneke Meyer, and others, organized the 1^st ^IBRO school in Africa which was hosted by the University of the North in 2000, in Pietersburg, South Africa. Terje remained a faithful supporter of IBRO, particularly in African activities, organizing ADHD workshops and establishing an ADHD network in Africa.

Terje had very close links with the University of the North, which became the University of Limpopo in 2005. He held his first workshop there in 1997 and was instrumental in obtaining funds from theNorwegian Universities' Committee for Development Research and Education (NUFU), which kept the ADHD project in Limpopo going for 12 years. Under his guidance a very important epidemiological study was completed, involving six different ethnic groups. More cross-cultural neuropsychological projects followed. He was co-supervisor for a number of PhD students, who will sadly miss his expert guidance and warm encouragement. In 2008 he organised a week long workshop on scientific writing in Oslo for the PhD students from Limpopo. This was an unforgettable event as for most of them it was their first time to travel outside South Africa. He will be sadly missed at this institution as a co-worker, teacher, and mentor, but above all for his warm-hearted interest and friendship.

Terje's approach to research was collaborative and multidisciplinary. He facilitated several collaborative projects across the world. In the academic year 2004-2005, he led an international research group at the Centre for Advanced Studies at the Norwegian Academy for Science and Letters which for so many of us was a key catalyst in generating new ideas and new lines of research thus enhancing our international collaborations (as illustrated by joint publications and the establishment of *Behavioral and Brain Functions *in 2005). In 2005 he also established the Oslo ADHD Group, integrating basic studies of anatomy, neurophysiology, neuropsychology, genetics, and behaviour. The group presented their research at a symposium in Oslo in 2010, together with researchers from Canada, South Africa, the US and Europe. Terje believed in collaboration, openness and willingness to share one's knowledge, insights and ideas in order to move science forward.

Always innovative and creative, Terje played a major role in designing experiments for children that were directly analogous to the behavioural studies with animals. Through building a response apparatus that could be operated by children in much the same way as that for the rats, behavioural responses could be analyzed in the same way across species [10]. Ultimately, behavioural patterns obtained with the rats could be validated against the clinical case of ADHD. The task was further developed into computer-based tasks that were used both in Norway and in South-Africa, showing that basic behavioural mechanisms operated the same way across cultures [11,12].

**Figure 2:**
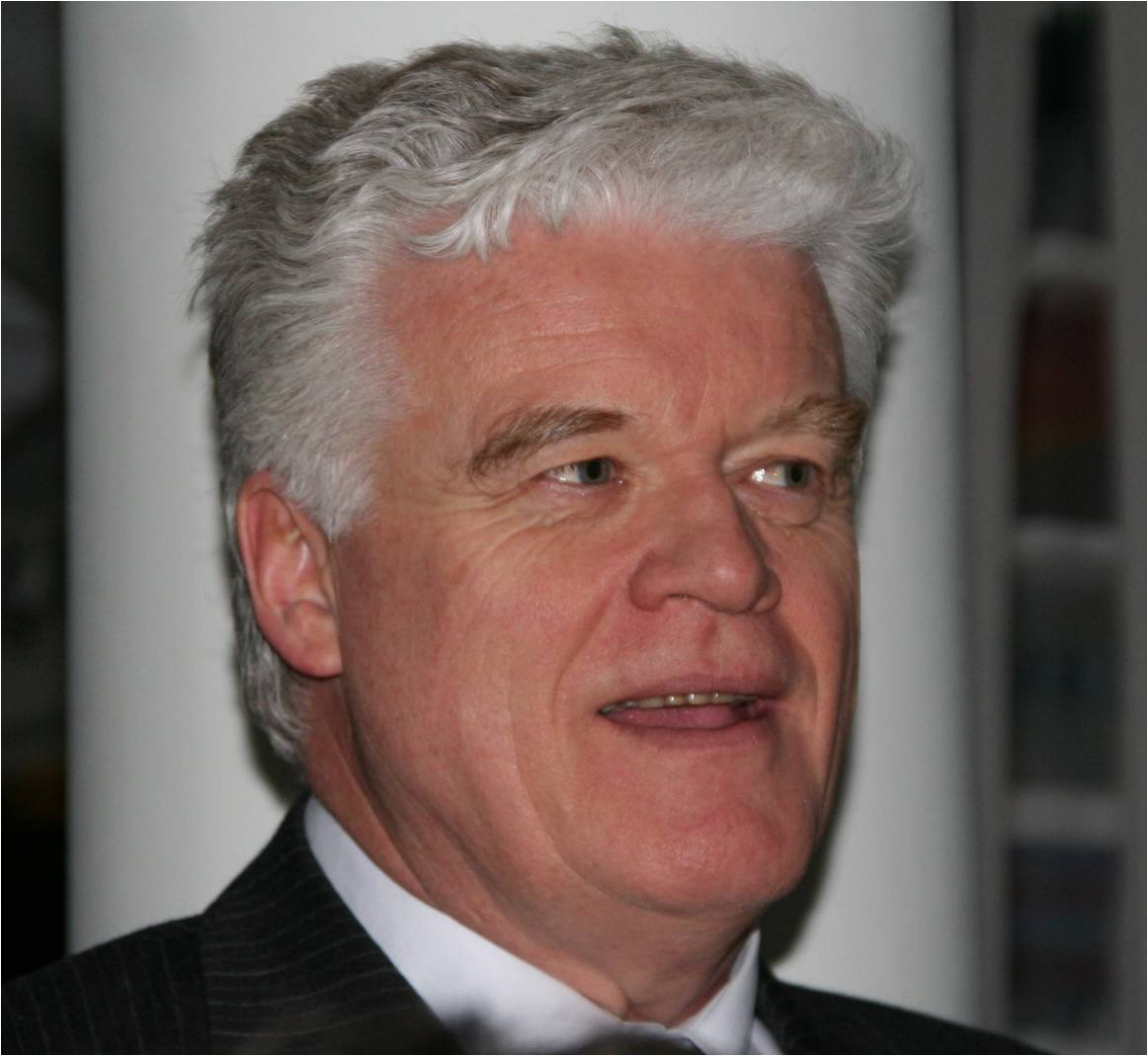
**Professor Terje Sagvolden**.

The list of contributions to the field of ADHD is impressive but fails to capture the passion with which he worked and interacted with his colleagues, who will sorely miss him (Figure 2).
